# 四川地区2008年-2013年3, 663例肺癌临床病理特征及流行趋势分析

**DOI:** 10.3779/j.issn.1009-3419.2016.02.03

**Published:** 2016-02-20

**Authors:** 爽 赵, 镭 李, 志新 邱, 越 程, 玉婷 景, 永召 周, 为民 李

**Affiliations:** 610041 成都，四川大学华西医院呼吸内科 Department of Respiratory Medicine, West China Hospital, Sichuan University, Chengdu 610041, China

**Keywords:** 肺肿瘤, 流行病学特征, 地区分布, 时间趋势, Lung neoplasms, Epidemiology, Regional distribution, Time trends

## Abstract

**背景与目的:**

肺癌作为全球男女性中致死率最高的肿瘤之一，严重威胁着人类的健康。近些年来，各个地区肺癌的发病率及死亡率也有逐年上升的趋势。本研究旨在分析四川地区肺癌患者的临床病理特征与流行趋势，了解肺癌地区与时间分布特点及差异。

**方法:**

收集2008年-2013年四川大学华西医院就诊的四川地区肺癌患者，分析川中、川南、川北、川西的患者临床病理特征及流行趋势。

**结果:**

纳入患者3, 663例（川中2, 378例，川南469例，川北584例，川西232例）。患者平均年龄59.6岁，各个地区患者平均年龄不同（*P* < 0.001），以川中最大，为61.0岁。患者以男性为主（68.4%），不同地区患者男性构成比存在差异（*P*=0.014），以川北最高，为73.3%。此外，各个地区的病理类型构成比也存在差异（*P*=0.014），腺癌和鳞癌都在川西构成比最高，分别为57.8%和30.2%，小细胞癌则在川北构成比最高（19.9%）。2013年腺癌与鳞癌构成比较2008年均有所降低，早期肺癌及淋巴结转移构成比有所上升。此外，不同地区肺癌患者3年生存率不同（*P*=0.021），以川西最低，仅为13.6%，可能与该地区采用姑息治疗患者比率高相关。

**结论:**

近六年于我院就诊的四川地区肺癌患者以川中为主，男性多见，中老年为甚，腺癌居多，且腺癌和鳞癌的构成比与六年前相比均有下降，早期肺癌及淋巴结转移构成比上升。不同地区肺癌患者的平均年龄、男性构成比、病理类型及3年生存率均有差异。

## Abstract (en)

**Background and objective:**

Lung cancer is the most common malignancy and is the leading cause of cancer-related death worldwide. Thus, this disease severely threatens human health. This study aims to identify the clinical epidemiology and histological characteristics of lung cancer patients in Sichuan areas.

**Methods:**

We enrolled 3, 761 lung-cancer patients who were identified as residents of Sichuan province and treated in West China Hospital from 2008 to 2013.

**Results:**

A total of 3, 663 patients from Central, Southern, North, and Western areas in Sichuan province, respectively, were enrolled. The average age of patients was 59.6 years, and patients were predominantly male (68.4%). Significant statistical differences were observed among the average age of patients, male, and pathological types in different regions (all *P* < 0.05). In addition, compared with the 2008 group, the 2013 group had lower rates of adenocarcinoma and squamous-cell carcinoma but higher rates of early-stage lung cancer and lymph-node metastasis. After a three-year follow-up of 1, 003 cases, results showed that the 3-year overall survival (OS) was not the same in different regions (*P*=0.021), and that the poorest OS was in Western Sichuan. This result may be related to the high rate of patients with palliative care.

**Conclusion:**

For the last six years, the patients with lung cancer in Sichuan were mainly from Central Sichuan, male patients, elder (age > 60 yr) patients, and with adenocarcinoma of the lung. Patients of 2013 had lower rates of adenocarcinoma and squamous-cell carcinoma but higher rates of early-stage lung cancer and lymph-node metastasis. Furthermore, the 3-year OS was not the same in different regions.

目前肺癌是全球发病率及致死率最高的肿瘤之一，尽管近些年治疗手段不断发展，但其5年生存率仅为17%^[1]^。在中国，近三年来，肺癌的死亡率上升了464.84%，这与吸烟及环境污染密切相关^[2]^。世界卫生组织（World Health Organization, WHO）预测中国到2025年时每年新发肺癌病例将超过100万，变成世界肺癌第一大国^[3]^。因此，了解肺癌的人群分布、地域及时间变化等流行病特征对肺癌的研究至关重要。本研究将对2008年-2013年四川大学华西医院收治的四川地区肺癌患者进行分析，了解肺癌临床特点及流行病学趋势，比较川中、川南、川北、川西肺癌患者临床病理特征及预后的差异，探索造成这些差异及分布的因素。

## 1 资料与方法

### 1.1 研究对象

本研究对象为2008年1月-2013年12月四川大学华西医院收治的四川地区肺癌患者。纳入标准：①初诊为原发性支气管肺癌患者，手术标本经病理学证实；②排除无病理学证据，临床资料不完整患者；③排除合并其他肿瘤或肺癌复治的患者。收集资料包括年龄、性别、病灶部位、病理学类型、临床分期、地区来源、诊断年份、吸烟史、淋巴结转移及远处转移情况。临床分期根据美国癌症联合委员会（American Joint Committee on Cancer, AJCC）的肿瘤-淋巴结-转移（tumor-node-metastasis, TNM）分期^[4]^、病理类型及分化程度按1999年WHO肺癌组织学类型分类标准^[5]^。并根据“2013年四川统计学年鉴^[6]^”及地理位置将四川地区划分为川中（成都、德阳、绵阳、遂宁、眉山、资阳），川南（自贡、泸州、内江、乐山、宜宾），川北（广元、南充、广安、达州、巴中）及川西（攀枝花、雅安、凉山彝族自治州、甘孜阿坝藏族羌族自治州）四个区域，其中以川中及川南经济较为发达，川西地区最为落后。总共纳入肺癌患者3, 663例，其中川中2, 378例，川南469例，川北584例，川西232例。对其进行3年随访，最终有1, 003例纳入生存分析。

### 1.2 统计学方法

采用SPSS 19.0进行统计学分析，使用χ^2^检验、*t*检验、秩和检验及*Fisher*确切概率进行组间比较。采用*Kaplan*-*Meier*生存曲线进行生存分析。采用GraphPad Prism进行图片制作。*P* < 0.05为差异有统计学意义。

## 2 结果

### 2.1 一般情况

如表 1所示，本研究总共纳入原发性支气管肺癌患者3, 663例，年龄19岁-93岁，平均59.65岁。其中男性2, 505例（占68.4%），女性1, 158例（31.9%）。纳入患者中，病理类型以腺癌为主，2, 032例，占55.5%；鳞癌980例（26.8%），小细胞癌595例（16.2%）。病灶部位以左肺多见（占54.8%）。临床分期中，早期肺癌（Ⅰ期-Ⅱ期）仅有644例（占17.5%），中晚期有3, 019例（82.5%）。此外，2, 426例（66.2%）肺癌患者在诊断时就有淋巴结转移，2, 161例（59.0%）有肝、脑、骨等远处转移。所有患者中有吸烟史的2, 002例（54.7%）。其中，鳞癌患者中吸烟者比率较其他病理类型高（*P*=0.009），约占59.2%。

**1:** 3, 663例肺癌患者临床病理特征 The clinicopathologic features of 3, 663 lung cancer patients

Characteristics	Data
Age (year, mean, range)	59.65 (19-93)
Gender	
Male	2, 505 (68.4%)
Female	1, 158 (31.9%)
Lesion location	
Left lobe	2, 009 (54.8%)
Right lobe	1, 600 (43.7%)
Diffuse	46 (1.3%)
Others	8 (0.2%)
Histology	
Adenocarcinoma	980 (26.8%)
Squamous cell carcinoma	2, 032 (55.5%)
Small cell lung cancer	595 (16.2%)
Others	56 (3.3%)
TNM stage	
Ⅰ-Ⅱ	644 (17.5%)
Ⅲ-Ⅳ	3, 019 (82.5%)
Smoking	
No	1, 661 (45.3%)
Yes	2, 002 (54.7%)
Lymphatic metastasis	
No	1, 237 (33.8%)
Yes	2, 426 (66.2%)
Distant metastasis	
No	1, 502 (41.0%)
Yes	2, 161 (59.0%)
TNM: tumor-node-metastasis.	

### 2.2 地区与肺癌患者临床特征分布

如表 2所示，四川根据地理位置被划分为四个地区，不同地区患者平均年龄不同（*P* < 0.000, 1），以川中最大，为61.0岁。所有地区肺癌患者均以男性为主，但各个地区男性构成比存在差异（*P*=0.014），其中川北最高（73.3%），川中最低（66.7%）。病理类型上看，各个地区均以腺癌为主，但是不同的病理类型在各个地区的构成比存不同（*P*=0.014），腺癌和鳞癌都在川西构成比最高，分别为57.8%和30.2%，小细胞癌则在川北构成比最高（19.9%）。然而，不同地区间肺癌的病灶部位、临床分期、吸烟史、淋巴结转移及远处转移不具有统计学差异（*P* > 0.05）。

**2:** 肺癌患者临床特征的地区分布特点 Region distribution characteristics of patients with lung cancer

Characteristics	Central. (*n*=2, 378)	Southern. (*n*=469)	North. (*n*=584)	Western. (*n*=232)	*P* value
Age (year, mean, range)	61.00 (19-93)	57.12 (24-82)	57.14 (19-82)	57.31 (23-81)	< 0.000, 1^*^
Gender					0.014^*^
Male	1, 586 (66.7%)	330 (70.4%)	428 (73.3%)	161 (69.4%)	
Female	792 (33.3%)	139 (29.6%)	156 (26.7%)	71 (30.6%)	
Lesion location					0.067
Left lobe	1, 353 (56.9%)	239 (51.0%)	301 (51.5%)	116 (50.0%)	
Right lobe	992 (41.7%)	220 (46.9%)	275 (47.1%)	113 (48.7%)	
Diffuse	26 (1.1%)	9 (1.9%)	8 (1.4%)	3 (1.3%)	
Others	7 (0.3%)	1 (0.2%)	0 (0)	0 (0)	
Histology					0.014^*^
ADC	624 (26.2%)	119 (25.4%)	167 (28.6%)	70 (30.2%)	
SCC	1, 352 (56.9%)	252 (53.7%)	294 (50.3%)	134 (57.8%)	
SCLC	364 (15.3%)	91 (19.4%)	116 (19.9%)	24 (10.3%)	
Others	38 (1.6%)	7 (1.5%)	7 (1.2%)	4 (1.7%)	
TNM stage					0.066
Ⅰ	235 (9.9%)	40 (8.5%)	60 (10.3%)	7 (3.0%)	
Ⅱ	194 (8.2%)	41 (8.7%)	52 (8.9%)	15 (6.5%)	
Ⅲ	554 (23.3%)	104 (22.2%)	136 (23.3%)	64 (27.6%)	
Ⅳ	1, 395 (58.7%)	284 (60.6%)	336 (57.5%)	146 (62.9%)	
Smoking					0.651
No	1, 064 (44.7%)	211 (45.0%)	276 (47.3%)	110 (47.4%)	
Yes	1, 314 (55.3%)	258 (55.0%)	308 (52.7%)	122 (52.6%)	
Lymphatic metastasis					0.642
No	819 (34.4%)	157 (33.5%)	186 (31.8%)	75 (32.3%)	
Yes	1, 559 (65.6%)	312 (66.5%)	398 (68.2%)	157 (67.7%)	
Distant metastasis					0.461
No	983 (41.3%)	185 (39.4%)	248 (42.5%)	86 (37.1%)	
Yes	1, 395 (58.7%)	284 (60.6%)	336 (57.5%)	146 (62.9%)	
ADC: Adenocarcinoma; SCC: Squamous cell carcinoma; SCLC: small cell lung cancer. Central.: Central Sichuan; Southern.: Southern Sichuan; North.: North Sichuan; Western.: Western Sichuan. ^*^P < 0.05.					

### 2.3 年份与肺癌患者临床特征分布

如表 3所示，纳入患者中，2008年124例，2009年810例，2010年893例，2011年868例，2012年526例，2013年442例。不同年份间肺癌患者的年龄、男性构成比、远处转移及吸烟史等情况均无明显统计学差异（*P* > 0.05）。然而，不同年份的肺癌患者病理类型不相同，与2008年相比，2013年腺癌与鳞癌构成比均有所降低（*P*=0.002）。早期（Ⅰ期-Ⅱ期）肺癌患者近5年构成比有所上升（*P*=0.044），2008年仅为13.7%，而2009年-2013年则波动在15.4%-19.9%。此外，伴有淋巴结转移的肺癌患者构成比近几年来亦有增加（*P* < 0.05）。

**3:** 肺癌患者临床特征的时间分布特点 Time distribution characteristics of patients with lung cancer

Year	2008	2009	2010	2011	2012	2013	*P* value
Total	124	810	893	868	526	442	
Age (year, Mean±SD)	59.37±12.00	59.80 ±12.12	59.71±11.65	59.43±11.71	59.59±12.16	59.89±12.16	0.144
Male proportion	83 (66.9%)	557 (68.7%)	600 (67.2%)	592 (68.2%)	372 (70.7%)	301 (68.1%)	0.823
Histological proportion							0.002^*^
SCC	32 (25.8%)	215 (26.5%)	264 (29.6%)	238 (27.4%)	135 (25.7%)	96 (21.7%)	
ADC	76 (61.3%)	443 (54.7%)	483 (54.1%)	507 (58.4%)	278 (52.9%)	245 (55.4%)	
Early stages (Ⅰ-Ⅱ)	17 (13.7%)	125 (15.4%)	163 (18.3%)	173 (19.9%)	98 (18.6%)	68 (15.4%)	0.044^*^
Lymphatic metastasis	81 (65.3%)	505 (62.3%)	607 (68.0%)	571 (65.8%)	348 (66.2%)	314 (71.0%)	0.044^*^
Distant metastasis	80 (64.5%)	472 (58.3%)	521 (58.3%)	507 (58.4%)	299 (56.8%)	282 (63.8%)	0.203
Smoking status	75 (60.5%)	436 (53.8%)	490 (54.9%)	473 (54.5%)	293 (55.7%)	235 (53.2%)	0.765
No.: number; SEM: standard error of mean. ^*^P < 0.05.							

### 2.4 不同地区肺癌患者的预后

对这3, 663例患者进行3年的随访，最终仅1, 003例患者获得生存资料。对其进行生存分析，如表 4及图 1所示，其中川中652例，川南130例，川北162例，川西59例。不同地区，肺癌患者的3年生存率不同（*P*=0.021），以川南的生存率最高（60.8%），川西最低（44.1%）。中位生存时间以川中及川南更长，分别是（30.030±0.889）个月和（31.785±1.688）个月；仍然以川西时间最短，仅（22.525±1.763）个月。进一步我们对不同地区可能影响预后的因素进行分析（表 5），发现不同地区间肺癌的诊断手段、患者的远处转移及临床分期情况均不具有统计学差异（*P* > 0.05）。而治疗方式上，手术、放疗、化疗及生物靶向治疗等在不同地区间亦不存在差异，仅有采用姑息治疗患者的比率有所不同（*P*=0.007），其中川西肺癌患者选择姑息治疗的比率最高，高达52.5%。

**4:** 四川不同地区肺癌患者的预后 The lung cancer patients' survival of different regions in Sichuan

Variables	Central.	Southern.	North.	Western.	*P* value
Total	652	130	162	59	
Death toll	522	94	133	51	
3-year survival rate	19.9%	27.7%	17.9%	13.6%	
Survival time	30.030±0.889	31.785±1.688	27.136±1.530	22.525±1.763	0.021

**1:**
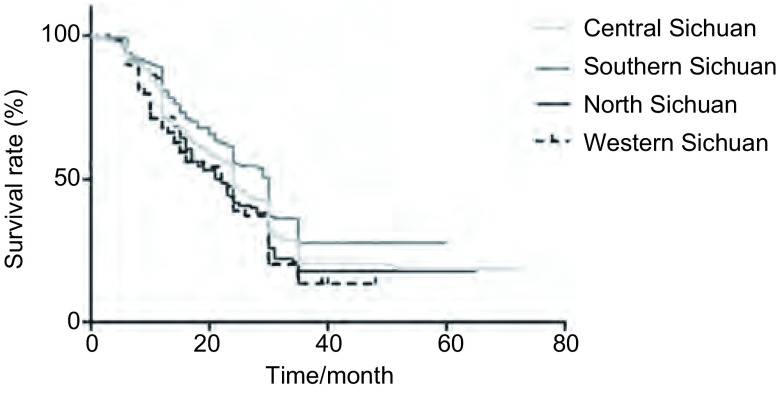
四川不同地区肺癌患者的生存曲线 Kaplan-Meier curves for lung cancer patients'survival in Sichuan different regions

**5:** 四川不同地区肺癌患者预后相关因素分析 The prognostic factors of lung cancer patients in Sichuan different regions

Variables	Central. (*n*=652)	Southern. (*n*=130)	North. (*n*=162)	Western. (*n*=59)	*P* value
Diagnosis methods					
Surgery	139	33	31	8	0.408
Bronchofiberscope	333	62	96	30	0.442
TNB	57	11	22	4	0.421
Sputum cytology	61	6	14	8	0.786
Pleural fluid cytology	96	17	19	11	0.834
Treatment methods					
Surgery	175	36	42	9	0.513
Radiotherapy	50	7	10	5	0.466
Chemotherapy	239	54	55	15	0.710
Biological target therapy	33	1	5	3	0.080
Palliative care	187	32	49	31	0.007^*^
Metastasis					
Distance	484	95	111	48	0.248
Lymph node	405	80	92	40	
TNM stage					
Ⅰ-Ⅱ	128	24	32	8	0.713
Ⅲ-Ⅳ	524	106	130	51	
TNB: transthoracic needle biopsy. ^*^P < 0.05.					

## 3 讨论

在全世界范围内，肺癌的发病率占所有恶性肿瘤的第二位，致死率是恶性肿瘤中的首位，其中男性为28%，女性为26%^[1]^。在美国所有恶性肿瘤中，肺癌的致死率男性为29%，女性为21%，均居于致死率之首^[7]^。陈万青等^[8]^对1988年-2005年我国10个地区进行抽样调查，发现这18年间，我国肺癌发病率是逐年上升的，年平均增长率为1.63%（男性是1.30%，女性是2.34%）。从性别上看，肺癌在全球发病率及死亡率均以男性为主。与1985年相比，目前的肺癌患者增加了51%，其中男性增加44%，女性增加77%^[9]^，表明女性肺癌的发病率正逐年上升，男女构成比逐年下降。而之所以男女性肺癌发病率不同可能是由于男女性的病理类型、吸烟率、被动吸烟率、基因易感性等存在差异^[10-14]^。但我们的研究发现，至我院就诊的四川地区肺癌患者男性构成比近六年来无明显差异，仍以男性为主。同时，我们的研究表明超过半数以上的肺癌患者有吸烟史，且鳞癌中吸烟者的比率较其他病理类型更高。虽然近六年吸烟肺癌患者构成比有下降趋势，但差异无统计学意义。吸烟仍是罹患肺癌的高危因素，尤其是在鳞癌中。Alberg等^[15]^研究表明主动吸烟与被动吸烟均增加肺癌的发病，且发病风险与初始烟龄成反比，吸烟量及吸烟年限成正比，吸烟与肺癌间是剂量-效应关系。Youlden等^[16]^发现对不同国家或地区的男女性的肺癌发病率及变化趋势进行观察，可一定程度上反映当地烟草流行阶段和程度。针对于不吸烟的人群，肺癌的发生可能与基因敏感性、环境及生活方式等相关。在台湾的一个对非吸烟汉族女性腺癌患者的研究^[17]^发现，5p15.33的突变与该人群肺癌的发生密切相关。

肺癌患者的5年生存率近几年没有明显改善，其中最为重要的原因是缺乏早期的诊断方法。本研究也发现肺癌患者在诊断时以中晚期为主，比率高达82.5%，且大多数患者均有淋巴结或者远处的转移，这对肺癌的治疗及预后产生了不良的影响。来自于荷兰西北地区的一项纳入13, 116例非小细胞肺癌（non-small cell lung cancer, NSCLC）患者的研究结果^[18]^与我们一致，均显示肺癌初诊时分期向晚期移行，早期肺癌（Ⅰ期-Ⅱ期）比例有所下降。可能是由于早期肺癌起病隐匿、无特异临床表现、患者不重视及当地诊疗水平有限等原因而延误诊断。我们的研究还发现近六年来，肺癌患者淋巴结转移的构成比成上升趋势，这种现象与医疗水平的进步密不可分。目前肺癌筛查及诊断方式越来越多样化，诊断的敏感性也是逐年在提高。所以应定期健康体检，尤其是胸部计算机断层扫描（computed tomography, CT）的筛查是尤为重要的，Henschke等^[19]^对1, 000例无症状的60岁以上的吸烟人群进行随访研究显示，低剂量螺旋CT与胸片相比，肺癌筛查的阳性率高5倍，而Ⅰ期肺癌筛查阳性率高6倍。对于已经有咳嗽、胸痛、咯血等症状的患者更应该及时就诊，行胸部CT、肿瘤标志物、纤维支气管镜等检查，尽快明确诊断，及时进行治疗。此外，更多从分子学层面上，关于肺癌诊断的特异性肿瘤标志物研究也是必要的。

不同地区的人群由于生活环境及生活方式不同，肺癌的流行病学特点也存在差异。我们根据" 2013四川统计学年鉴" 及地理位置将四川划分为四个区域，研究发现至华西医院就诊患者以川中为主，占所有纳入人群的64.9%，而川西的最少，仅占6.3%，可能与川中地区人群经济条件、健康意识、就诊便捷程度较其他地区更好等有关。此外，川中地区患者的平均年龄也较其他地区高，同样可能是由于该地区的经济条件与健康政策较好，使肺癌的发病年龄有所推迟，但具体原因需要进一步探究。而本研究还发现不同地区患者男性构成比、病理类型构成比均存在差异，则可能与该地区人群生活环境、污染程度、饮食习惯及生活方式相关。美国癌症协会（American Cancer Society, ACS）的资料显示，大气中的细颗粒物（particulate matter, PM）2.5的年均浓度每上升10 μg/m^3^，人群中肺癌的病死率则升高8%^[20]^。该团队进一步对1, 100例非吸烟肺癌患者进行26年的随访研究，发现PM2.5每增加10 μg/m^3^，肺癌患者的死亡率增加15%-27%^[21]^。在台湾的一项对空气中的六种污染物气体（SO_2_、CO、NO_2_、NO、O_3_和PM10）的研究^[22]^表明，SO_2_可明显增加肺腺癌及鳞癌的患病风险，且与SO_2_的浓度呈正相关，而其他五种污染气体对增加肺腺癌及鳞癌患病风险不明显。因此，空气污染与肺癌的发生密切相关。关于饮食对肺癌影响的研究也有许多，其中有一项针对1, 674例肺癌患者与1, 735位健康人的研究^[23]^显示，饮食中摄入较多植物雌激素、木酚素、大豆异黄酮、植物固醇等，可以降低健康人患肺癌的风险及改善肺癌患者的预后，主要是由于饮食的高抗氧化剂及微量营养素可减少脱氧核糖核酸（deoxyribonucleic acid, DNA）的氧化损伤，从而达到预防肿瘤的效果。Kubik等^[24, 25]^发现吸烟的女性增加奶制品和葡萄酒的摄入，适当的锻炼可减少肺癌的风险；非吸烟妇女多饮红茶可降低肺癌的发生。目前为止，人们已经开始关注环境、饮食等与肺癌发病率的关系，但现仍缺乏设计严密、大样本的流行病学研究资料。

我国30个市县的肿瘤登记资料示，男女性中肺癌死亡率较高的城市有上海、北京、哈尔滨、鞍山等，地理位置上出现由北向南、由东向西逐步下降的趋势^[26]^。此外，该研究发现城市肺癌的死亡率高于农村，且与城市的规模正相关，但农村的发病率近年亦有上升的趋势。本研究表明四川不同地区，肺癌患者的3年生存率不同。以川南的生存率最高，川西最低，中位生存时间以川中及川南更长。川中及川南地区的经济较另两个地区发达，城镇化率也较高。而川西地区地理位置距我院最远，经济水平相对其他区域落后，少数民族所占比率也较高。该地区患者至我院就诊人数相比其他区域少，而就诊的患者选择姑息保守治疗的比例也最高。这些都与该地区患者3年生存率较低密切相关。其他因素如人群、环境、遗传敏感性等的影响，需要更多大样本量的研究进行证实。

综上所述，近六年于我院就诊的四川地区肺癌患者以川中地区为主，男性多见，中老年为甚，腺癌居多，且腺癌和鳞癌的构成比与六年前相比均有下降，而早期肺癌及淋巴结转移构成比上升。不同地区肺癌患者的平均年龄、男性构成比、病理类型分布均有差异。川中及川南地区肺癌3年生存率较高，而川西最低，可能与该地区采用姑息治疗患者比率高相关。了解肺癌的人群、地域分布及时间变化等流行病特征对肺癌的研究至关重要，为肺癌的诊治及预防提供了依据。因此，我们应该加大戒烟教育力度，提高人群自觉戒烟意识，实现公共场合无烟化；在城市建设的同时，加强环境污染治理，减少污染物排放；加强人群的自我健康管理意识，尤其是对高危人群，更应普及健康体检，加大肺癌筛查的力度^[27]^。真正做到早筛查，早诊断，早治疗。相信在政府部门、医疗卫生机构及社会群体的共同努力下，人们对肺癌的防治工作将会取得更大成果。
